# Dosimetric Comparison of Proton Radiation Therapy, Volumetric Modulated Arc Therapy, and Three-Dimensional Conformal Radiotherapy Based on Intracranial Tumor Location

**DOI:** 10.3390/cancers10110401

**Published:** 2018-10-26

**Authors:** Sebastian Adeberg, Semi B. Harrabi, Nina Bougatf, Vivek Verma, Paul Windisch, Denise Bernhardt, Stephanie E. Combs, Klaus Herfarth, Juergen Debus, Stefan Rieken

**Affiliations:** 1 Heidelberg Institute of Radiation Oncology (HIRO) and DKTK Site Heidelberg, Im Neuenheimer Feld 400, 69120 Heidelberg, Germany; Semi.harrabi@med.uni-heidelberg.de (S.B.H.); nina.bougatf@med.uni-heidelberg.de (N.B.); Paul.windisch@med.uni-heidelberg.de (P.W.); denise.bernhardt@med.uni-heidelberg.de (D.B.); Klaus.herfarth@med.uni-heidelberg.de (K.H.); Juergen.debus@med.uni-heidelberg.de (J.D.); Stefan.rieken@med.uni-heidelberg.de (S.R.); 2 Heidelberg Ion-Beam Therapy Center (HIT), Im Neuenheimer Feld 450, 69120 Heidelberg, Germany; 3 Department of Radiation Oncology, University Hospital Heidelberg, Im Neuenheimer Feld 400, 69120 Heidelberg, Germany; 4 Clinical Cooperation Unit Radiation Oncology, German Cancer Research Center (DKFZ), Im Neuenheimer Feld 280, 69120 Heidelberg, Germany; 5 Department of Radiation Oncology, Allegheny General Hospital, Pittsburgh, PA 15212, USA; vivek333@gmail.com; 6 Department of Radiation Oncology, Technische Universität München, Ismaninger Straße 22, 81675 München, Germany; Stephanie.combs@tum.de; 7 Department of Radiation Sciences (DRS), Institut für Innovative Radiotherapie (iRT), Helmholtz Zentrum München, Ingolstädter Landstraße 1, 85764 Neuherberg, Germany

**Keywords:** proton therapy, IMPT, VMAT, IMRT, 3D conformal radiotherapy, glioma, glioblastoma, astrocytoma, craniopharyngioma, neurocognition, cost-effectiveness

## Abstract

(1) Background: Selecting patients that will benefit the most from proton radiotherapy (PRT) is of major importance. This study sought to assess dose reductions to numerous organs-at-risk (OARs) with PRT, as compared to three-dimensional conformal radiotherapy (3DCRT) and volumetric-modulated arc therapy (VMAT), as a function of tumor location. (2) Materials/Methods: Patients with intracranial neoplasms (all treated with PRT) were stratified into five location-based groups (frontal, suprasellar, temporal, parietal, posterior cranial fossa; *n* = 10 per group). Each patient was re-planned for 3DCRT and intensity-modulated radiotherapy (IMRT) using similar methodology, including the originally planned target and organ-at-risk (OAR) dose constraints. (3) Results: In parietal tumors, PRT showed the most pronounced dose reductions. PRT lowered doses to nearly every OAR, most notably the optical system and several contralateral structures (subventricular zone, thalamus, hippocampus). For frontal lobe cases, the greatest relative dose reductions in mean dose (D_mean)_ with PRT were to the infratentorial normal brain, contralateral hippocampus, brainstem, pituitary gland and contralateral optic nerve. For suprasellar lesions, PRT afforded the greatest relative D_mean_ reductions to the infratentorial brain, supratentorial brain, and the whole brain. Similar results could be observed in temporal and posterior cranial fossa disease. (4) Conclusions: The effectiveness and degree of PRT dose-sparing to various OARs depends on intracranial tumor location. These data will help to refine selection of patients receiving PRT, cost-effectiveness, and future clinical toxicity assessment.

## 1. Introduction

Radiotherapy (RT) for intracranial neoplasms, many of which are benign and/or have good long-term prognosis, is inherently associated with a risk of damaging normal brain parenchyma and causing late toxicities [1,2]. The latest effort to deliver highly conformal RT and potentially decrease chronic adverse effects of cerebral irradiation in appropriate patients is manifested by the rapid rise of proton radiotherapy (PRT).

As compared to photons, PRT offers a unique dose deposition known as the Bragg peak, before and beyond which there is only limited dose absorption [3] in the normal tissue, and therefore only limited effects in non-target tissue. As a result, the major advantage of PRT over photon techniques such as three-dimensional conformal RT (3DCRT) and volumetric-modulated arc therapy (VMAT) is the step dose gradient (fall off) that provides a low dose profile in the entrance and exit path of the particles. Data from multiple tumor sites has demonstrated superior dosimetry with PRT over photon modalities [4,5,6,7,8,9], which is also valid for intracerebral neoplasms [10,11].

However, a central question facing radiation oncology is the assessment of which patients will benefit most from PRT. It has been posited by Zietman et al. that PRT may be most advantageous based on the location and anatomic considerations of the tumor with surrounding tissue [12]. When applied to intracranial malignancies, it is important to consider the location of primary tumors to be irradiated with PRT. Here, dosimetric differences as compared to photon-based techniques vary greatly based on location. For instance, tumors abutting the optic apparatus or the brainstem, especially if dose-escalation is warranted, may show the most striking dosimetric differences between PRT and photons. Conversely, it is likely that tumors that are relatively away from dose-limiting structures may have negligible dosimetric differences.

To this extent, we performed a planning study of patients with intracranial malignancies based on five different locations in order to assess the magnitude of dosimetric differences between PRT and photon-based modalities in each location. Implications of these results include more refined selection of patients receiving PRT, cost-effectiveness, and future clinical toxicity assessment.

## 2. Results

Each cohort comprised 10 patients for comparison, minus one patient (frontal lobe) for whom a clinically reasonable 3DCRT plan could not be generated. Patient characteristics are depicted in Table 1. Target coverage was similar for all locations between all three modalities.

**Figure 1:**
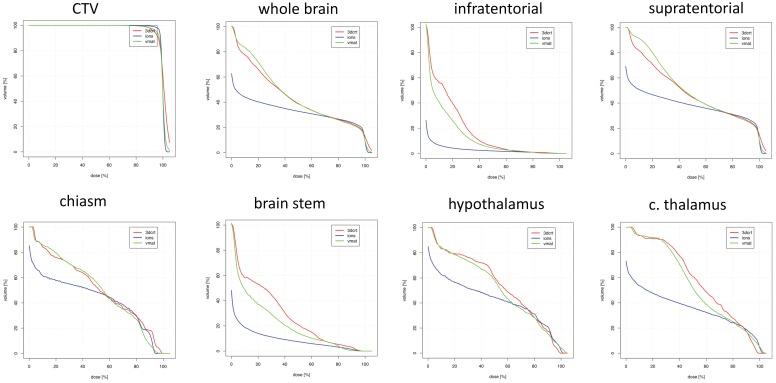
Dosimetric comparison: clinical target volume coverage and dose volume histogram curves of the organs-at-risk in patients with frontal tumor localization. Curves demonstrate median dose values of all patients with frontal tumor localization. Intensity-modulated proton therapy (IMPT/PRT; blue line) vs. volumetric-modulated arc therapy (VMAT; green line) vs. three-dimensional conformal radiotherapy (3DCRT; red line). CTV: clinical target volume; c.: contralateral.

Table 2 and Figure 1 display mean doses to targets and organs-at-risk (OARs) for frontal lobe cases. Most notably, PRT afforded the greatest relative dose reductions in D_mean_ to the contralateral hippocampus, optic nerve, brainstem, and infratentorial normal brain. This was at the expense of structures with D_mean_ increases with PRT such as the ipsilateral optic nerve. Most maximum doses were comparable (within ± 10% dose difference of photon techniques) for various OARs.

**Figure 2:**
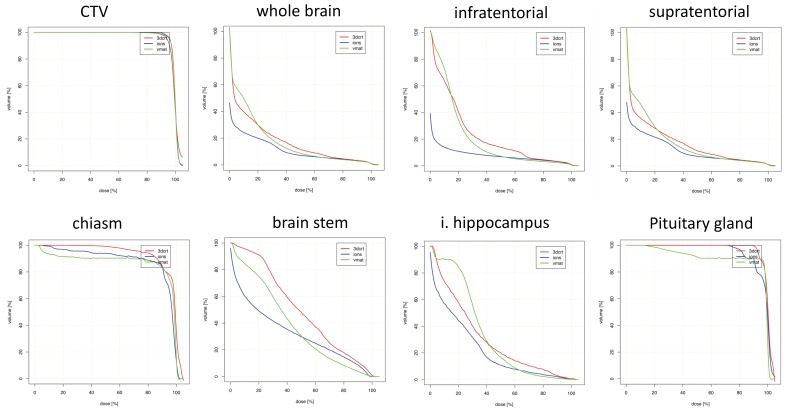
Dosimetric comparison: clinical target volume coverage and dose volume histogram curves of the organs-at-risk in patients with suprasellar tumor localization. Curves demonstrate median dose values of all patients with suprasellar tumor localization. Intensity-modulated proton therapy (IMPT/PRT; blue line) vs. volumetric-modulated arc therapy (VMAT; green line) vs. three-dimensional conformal radiotherapy (3DCRT; red line). CTV: clinical target volume; i.: ipsilateral.

Figures for suprasellar lesions are illustrated in Table 3 and Figure 2. Maximum doses were again comparable for most OARs. The greatest D_mean_ reductions with PRT were observed for whole brain, supratentorial brain and infratentorial brain, with no large dose increases by utilizing PRT.

**Figure 3:**
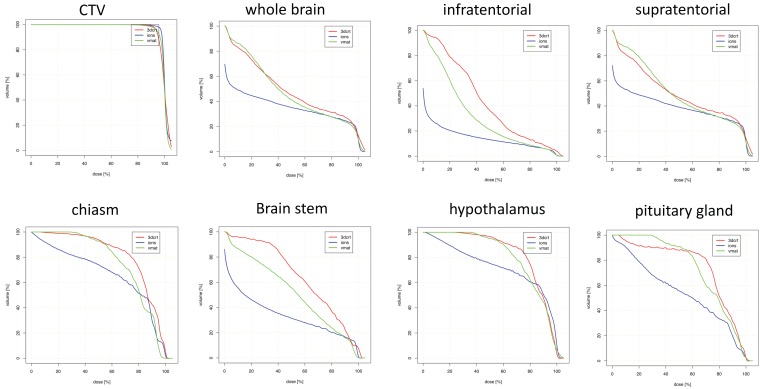
Dosimetric comparison: clinical target volume coverage and dose volume histogram curves of the organs-at-risk in patients with temporal tumor localization. Curves demonstrate median dose values of all patients with temporal tumor localization. Intensity-modulated proton therapy (IMPT/PRT; blue line) vs. volumetric-modulated arc therapy (VMAT; green line) vs. three-dimensional conformal radiotherapy (3DCRT; red line). CTV: clinical target volume.

Table 4 and Figure 3 show data regarding temporal tumors. Maximum doses were also similar for most OARs. OARs for which PRT led to the greatest mean dose reductions were the contralateral hippocampus and thalamus.

**Figure 4:**
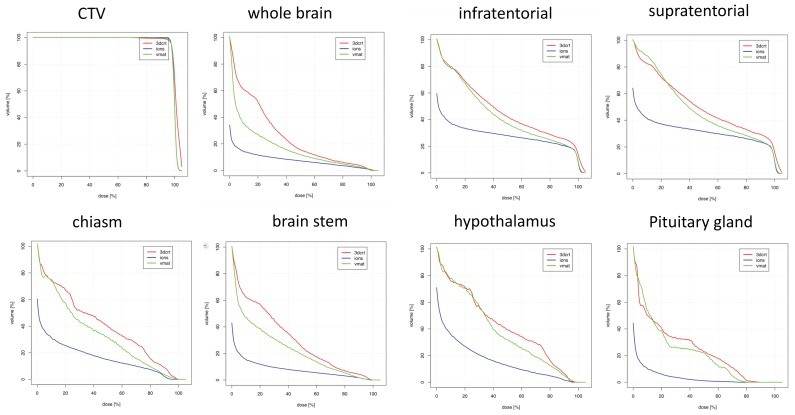
Dosimetric comparison: clinical target volume coverage and dose volume histogram curves of the organs-at-risk in patients with parietal tumor localization. Curves demonstrate median dose values of all patients with temporal tumor localization. Intensity-modulated proton therapy (IMPT/PRT; blue line) vs. volumetric-modulated arc therapy (VMAT; green line) vs. three-dimensional conformal radiotherapy (3DCRT; red line). CTV: clinical target volume.

Parietal targets displayed the most notable dose reductions with PRT (Table 5 and Figure 4). This included multiple maximum dose (D_max)_ values for several OARs, including bilateral optic nerves, brainstem, and chiasm. Decreased mean doses to nearly every OAR was observed with PRT, most notably bilateral optic nerves and several contralateral structures (subventricular zone (SVZ), thalamus, hippocampus).

**Figure 5:**
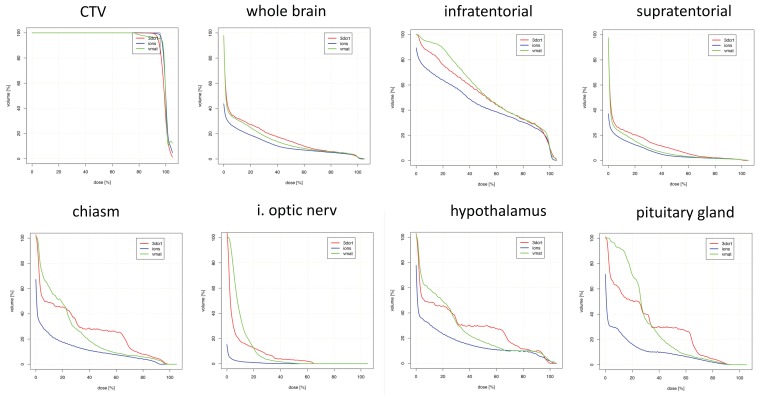
Dosimetric comparison: clinical target volume coverage and dose volume histogram curves of the organs-at-risk in patients with tumor localization in the posterior cranial fossa. Curves demonstrate median dose values of all patients with posterior cranial fossa tumor localization. Intensity-modulated proton therapy (IMPT/PRT; blue line) vs. volumetric-modulated arc therapy (VMAT; green line) vs. three-dimensional conformal radiotherapy (3DCRT; red line). CTV: clinical target volume; i.: ipsilateral.

Lastly, Table 6 and Figure 5 display values for posterior cranial fossa disease. D_max_ values were comparable or slightly decreased with PRT for all OARs except the brainstem. D_mean_ reductions were highest in bilateral optic nerves, and the anatomical area corresponding to the hypothalamus/pituitary/chiasm. There were non-significant relative increases in D_mean_ for bilateral thalami and SVZ, however, along with the ipsilateral hippocampus.

Comparable CTV coverage could be achieved in all three treatment modalities. In one patient, no reasonable 3DCRT plan could be generated due to proximity to critical OARs. This plan was excluded from the analysis. The inhomogeneity coefficient (IC) and homogeneity index (HI) were mostly equivalent or superior in the proton plans as compared to 3DCRT and VMAT, without reaching statistical significance, as depicted in Table 7.

## 3. Discussion

Our results offer overarching summaries that the PRT dose-sparing effect on various OARs is fundamentally dependent on tumor location. In other words, acknowledging the reliance on treatment planning techniques, PRT is more adept at sparing certain OARs depending on the location of primary disease. These data help to improve refined selection of patients receiving PRT, cost-effectiveness, and future clinical toxicity assessment.

When creating a treatment plan for any patient, regardless of technique or modality, it is impossible to spare every OAR to the lowest level possible. Rather, depending on which OARs are prioritized for a particular patient with a lesion in a given location (e.g., in terms of pre-existing comorbidities, life expectancy, and cost of managing late toxicities), this study may assist in evaluating whether PRT is indeed the most effective option. We acknowledge the limited applicability to other cases undergoing different treatment planning techniques, protocols, beam arrangements, and/or optimizations; nevertheless, these results can improve patient selection. For instance, if hippocampal sparing to the fullest degree is desired in a high-functioning young patient with parietal low-grade glioma, PRT may indeed be an appropriate option. The same may not be true if the tumor is in the posterior cranial fossa, and such cases may be equivalently served with state-of-the art IMRT techniques. The addition of these results to patient selection for PRT have large implications on cost-effectiveness, as detailed below.

Based on our results, broad generalizations based on location include that PRT provides the most optimal dose-sparing to OARs located at intermediate and farther distances from the target. In individual cases, PRT can lower OAR D_mean_ and D_max_ in proximity to the target; however, this cannot be displayed in a heterogenous cohort or even enable sufficient dose coverage of the target volume in proximity to critical OARs. In this context, in temporal tumors, excellent D_mean_ reductions are observed for the contralateral, but not ipsilateral, hippocampus. Nevertheless, although it is acknowledged that clinical and economic ramifications of dosimetric sparing of most intracerebral organs is currently unclear, it is likely that organs in the “intermediate distance” range could experience the most “clinically meaningful” toxicity reductions from improved dosimetry in terms of decreased D_mean_ with PRT. This is because dose reductions offered by PRT to distant organs is largely of overall low magnitude, on account of the already lower doses reaching farther anatomic locations. Another major theme herein is that PRT can effectively reduce integral brain doses (e.g., to normal whole brain tissue, or that supra/infratentorially), which some studies have postulated to relate to secondary malignancies [13]. However, it must be cautioned that the notion of “clinically meaningful” toxicity reductions are currently theoretical and not supported by prospective randomized evidence thus far [14].

Importantly, though the main goal of this study was to separate disease based on anatomical location, it is essential to recognize that achievable OAR doses are heavily dependent on particular locations within one of the five subsites delineated herein. For instance, a midline frontal lobe lesion and a well-lateralized one may have starkly different (contralateral) OAR doses (and dose-sparing), although both are still categorized in the same location group for this investigation. Hence, to that extent, our results do admittedly generalize within a group, but the overall conclusion should be that OAR sparing potential should still be taken on an individualized basis. The authors focused mainly on the comparisons of PRT versus other techniques. However, comparisons of 3DCRT versus VMAT were also performed (Table 7). Here, no striking differences could be proven, which could be due to the high standard of variation of all five cohorts. This could lead to a demarcation of finer differences in dose reduction of −1% to −50% that did not reach statistical significance in the majority of calculations (Table 2, Table 3, Table 4, Table 5 and Table 6).

The implications of these results include cost-effectiveness of PRT [15]. Though a complete discussion is beyond the scope of this article, it should be mentioned that in longer-term survivors, there may be cost-savings associated with a decrease in dose to several OARs, although a link between dosimetry and clinical toxicity reductions remains to be proven. For instance, preservation of memory and quality of life from decreased hippocampal doses (a focus of the Radiation Therapy Oncology Group 0933 trial [16]) are both associated with economic cost reductions. Similarly, it may be extrapolated from our findings that proton irradiation of tumors in any location may have the highest likelihood of having cost-effective PRT delivery, depending on the particular location within an anatomical subsite as discussed above. Further data are needed in order to corroborate this notion, however.

Our results complement other series that performed treatment planning comparisons in different entities. Fuss and colleagues performed a dosimetric comparison for optic pathway glioma, wherein PRT could reduce the D_max_ to the contralateral optic nerve by almost 50%; chiasm and pituitary gland doses were also reduced significantly using PRT [17]. Pituitary dysfunction and secondary hypopituitarism are well known late effects of cranial irradiation [18,19], which becomes more relevant and prevalent in long-term survivors. The prevention of second malignancies is of minor importance in high-grade gliomas due to the still limited life expectancy, but is of interest in other intracranial tumors [20,21]. In centrally localized craniopharyngioma, PRT was superior to IMRT when comparing radiation dose exposure to the contralateral SVZ and hippocampus [22]. When discussing the preservation of neuronal function after radiation therapy, the dosimetric advantages of PRT for the whole brain, hippocampus, temporal lobes, and sensory organs are easily apparent. Karunamuni and colleagues were able to discern a dose-dependent thinning of the cerebral cortex of 0.0033 mm/Gy with a pronounced effect in the temporal lobes and limbic cortex [23]. It seems obvious that the degree of radiation-related neurocognitive dysfunction is a function of the exposed treatment volume and dose to the critical organs.

Though difficult to extrapolate these results to children, the advantages become even more relevant in this population. Declines in intelligence quotient (IQ), processing speed, and fine motor skills have been reported after conformal radiation therapy [24,25,26]. To gain a better view on this topic, we performed a more detailed analysis of organs responsible for neurocognitive performance like the hypothalamus, the hippocampus, and the SVZ. The hippocampus and the bilateral SVZ are known to harbor neural progenitor cells [27,28], that are assumed to contribute to neurogenesis and injury repair with their self-renewing capacities [29,30], which could play a major role in the origin of neurocognitive impairment. However, the role of radiotherapeutic dose to the stem-cell niches is discussed controversial in the community [31]. In glioblastoma, increased doses to the SVZ correlated with improved outcomes [32,33], suggesting tumor propagation and regeneration by cancer stem cells (CSCs) localized in the stem-cell niches.

The limitations of this analysis, in addition to the retrospective nature and relatively small sample size, include institutional-related nuances of treatment planning, beam arrangement, treatment technique, and optimization. It is also acknowledged that mean doses were most focused upon herein, but other parameters are undoubtedly important as well, depending on the level of correlative data available, as well as the type of OAR. A major limitation is that OAR doses are enormously dependent on priorities and weights of optimization and treatment planning, thus limiting the applicability of this and any other dosimetric study. The inclusion of different tumor types is of less consequence in a planning study similar to this, although outliers in size cannot be ignored. Retrospective contouring of several OARs that were not initially used for dose calculations is also acknowledged. Moreover, although location is not a discrete variable and tumors were lumped into specific categories, it is also not excluded that disease in different locations within each of the five groups may result in different results than those observed here. As such, further experiences are needed to validate these conclusions.

## 4. Materials and Methods

### 4.1. Patient Selection

Fifty non-consecutive patients with primary brain tumors underwent proton radiotherapy at the Heidelberg Ion-Beam Therapy Center (HIT), University Hospital Heidelberg, between 2012 and 2015. Patients were included in the evaluation if the following criteria were met: primary intracranial malignancy, completion of PRT therapy, adequate magnetic resonance imaging (MRI) imaging, and available raw data of the RT plan. Patient characteristics are depicted in Table 1. Patients were classified according to the location of the tumor epicenter: frontal, suprasellar, temporal, parietal, and posterior cranial fossa.

### 4.2. Tumor and Organs-At-Risk (OARs) Delineation

Target volumes and OARs were delineated on original treatment planning computed tomography (CT) scans on axial views. Pre-therapeutic MRI with contrast-enhanced T1 and T2-fluid-attenuated inversion recovery (FLAIR) sequences were fused for purposes of target delineation. Target volume contouring techniques and methodology were applied as described earlier [10,11]. Generally, the gross tumor volume (GTV) represents the macroscopic visual tumor and/or the resection cavity. The clinical target volume (CTV) comprises the high risk area with respect to microscopic spread, but respecting anatomical barriers. For example, in glioblastoma, according to the European guidelines, a 2 cm margin I is generated on top of the GTV to receive the CTV. An additional margin of 2–5 mm is added to the CTV to receive the planning target volume (PTV). The margin is highly depended on the immobilization aids, image-guidance and radiation technique. For photon and particle therapy planning, OARs and target volumes were contoured using the Siemens Dosimetrist and Oncologist software (Siemens, Erlangen, Germany). Critical structures for neurogenesis and neuronal functions were contoured retrospectively. Tumor key areas were determined to set laterality and localization.

The brainstem, subventricular zone (SVZ), hippocampus, hypothalamus, and thalamus were contoured as previously reported [34,35,36,37]. Other neuronal structures, including the pituitary gland and optic system, were contoured as per previous reports [38] and the RTOG (Radiation Therapy Oncology Group) contouring atlas. Tolerance doses of OARs were based on QUANTEC (Quantitative Analyses of Normal Tissue Effects in the Clinic) guidelines [39,40,41]. Additional OARs were not taken into consideration at the time of treatment planning and delivery. For normal infratentorial, supratentorial and whole brain volumes, the PTV volumes were subtracted.

### 4.3. Treatment Planning

A 3DCRT, VMAT, and PRT plan were established on the original initial planning CT datasets, and dose recalculation was performed with the initial dose constraints and parameters. Re-planning on the Oncentra MasterPlan^®^ (Nucletron, Columbia, SC, USA) planning system for photon plans and the Syngo PT Planning (Siemens, Erlangen, Germany) for proton plans was carried out by a single experienced radiation therapist. Proton beams were delivered as Intensity-Modulated Proton Beam Radiotherapy (IMPT). Ion techniques using two to three coplanar or non-coplanar beams were applied by the horizontal beam-line or the gantry. Proton beam delivery used a pencil-beam full-width-at-half-maximum (FWHM) of 10 mm. For all plans, CTVs received at least 95% of the prescribed dose. Additional OARs were not considered as an avoidance structure in all modalities.

### 4.4. Comparative Evaluation of Treatment Plans

The target volume coverage and doses to relevant OARs were evaluated for PRT, 3DCRT and VMAT. Plans were evaluated using dose volume histograms (DVHs). A DVH represents a histogram relating dose tissue volume in treatment planning for radiation therapy. The homogeneity index (HI) and inhomogeneity coefficient (IC) were used to describe the dose distribution of the CTV.

The HI is supposed to be as close to 0 as possible and describes the homogeneity of the target volume [42]. Dp = prescribed dose; D_5_ and D_95_ = minimum dose in 5% and 95% of the target volume. IC assesses the target dose distribution. Here, higher values are associated with a greater variability in the target volume [43]. D_mean_, D_min_ and D_max_ = average, minimum and maximum target volume doses.

$$\;\mathrm{Homogeneity}\;\mathrm{index}\;\left(\mathrm{HI}\right)\;=\frac{D_{5}-D_{95}}{\mathrm{Dp}}\;\times \;100\;$$

$$\;\mathrm{Inhomogeneity}\;\mathrm{coefficient}\;\left(\mathrm{IC}\right)\;=\;\frac{\mathrm{Dmax}-\mathrm{Dmin}}{\mathrm{Dmean}}\;$$

### 4.5. Data and Statistical Analysis

All data was collected in a central research database [44]. To achieve maximal comparability, dosimetric analysis for all techniques was performed on a directly connected analysis platform [45] with customized analytic tools. Custom-fitted workflows were used to analyze dose data across all patients automatically.

In order to evaluate statistical differences among treatment plans, the Wilcoxon-signed rank test was applied with corresponding two-sided 95% confidence intervals using SigmaPlot™ (Systat Software GmbH, Erkrath, Germany) software. *p*-values smaller than 0.05 were considered statistically significant.

### 4.6. Ethics

The study was approved by the ethics committee of the University of Heidelberg, Germany (S-421/2015).

## 5. Conclusions

The effectiveness and degree of PRT dose-sparing to various OARs depends on intracranial tumor location. These data will help to refine selection of patients receiving PRT, cost-effectiveness, and future clinical toxicity assessment.

## Figures and Tables

**Table 1:** Patient characteristics. Numbers in brackets represent percentages and refer to the absolute values in front.

Cofactors	All	Frontal	Temporal	Parietal	Posterior Cranial Fossa	Suprasellar
	*n* = 50	*n* = 10	*n* = 10	*n* = 10	*n* = 10	*n* = 10
Gender						
Male	32 (64)	5 (50)	5 (50)	7 (70)	7 (70)	8 (80)
Female	18 (36)	5 (50)	5 (50)	3 (30)	3 (30)	2 (20)
Median age (range)	21 (4–62)	32 (15–37)	28 (16–62)	49 (27–55)	16 (5–28)	13 (4–6)
Diagnosis						
Glioma WHO °I	10 (20)	0 (0)	3 (30)	0 (0)	7 (70)	0 (0)
Glioma WHO °II	14 (28)	3 (30)	3 (30)	6 (60)	2 (20)	0 (0)
Glioma WHO °III	11 (22)	6 (60)	3 (30)	2 (20)	0 (0)	0 (0)
Glioma WHO °IV	5 (10)	1 (10)	1 (10)	2(20)	1 (10)	0 (0)
Craniopharyngioma	10 (20)	0 (0)	0 (0)	0 (0)	0 (0)	10 (100)
CTV (range) in cc.	125 (6–642)	262 (108–595)	228 (18–642)	235 (15–507)	28 (6–130)	35 (11–121)
PTV (range) in cc.	171 (11–744)	336 (149–734)	292 (32–744	293 (19–636)	46(11–162)	37 (18–159)

Abbreviations: WHO: world health organization; CTV: clinical target volume; PTV planning target volume.

**Table 2:** Median values for mean and maximum doses to target volumes and organs-at-risk by radiotherapy modality for frontal tumors.

Modality	CTV	PTV	iON	cON	BS	Csm	iSVZ	cSVZ	iThal	cThal	iHip	cHip	Pitu	HT	WB	IT	ST
MEAN DOSE																	
3DCRT	100.9	99.9	33.9	8.5	17.5	43.0	67.7	54.3	92.4	66.7	56.6	25.6	11.3	52.0	46.9	20.1	50.6
VMAT	99.8	98.2	50.2	19.9	12.8	59.3	73.6	49.5	84.4	56.0	55.2	23.7	43.8	57.4	47.5	10.2	54.7
PRT	99.8	98.9	55.9	6.4	7.1	53.4	68.9	22.3	80.5	24.3	53.6	1.2	21.4	44.2	34.3	1.8	39.9
% change, PRT vs. 3DCRT	−1	−1	−12	**−98**	**−75**	−30	−3	−71	−4	−56	+8	**−100**	−77	−29	−33	**−91**	−31
% change, PRT vs. VMAT	0	+1	+11	**−83**	**−58**	−11	−2	−59	−1	−30	−2	**−100**	**−68**	−20	−28	**−82**	−26
MAXIMUM DOSE																	
3DCRT	108.7	108.7	42.5	48.5	86.9	78.2	99.2	103.2	97.0	99.8	93.2	94.8	29.9	89.7	108.7	88.2	108.7
VMAT	107.4	107.4	90.2	52.6	84.8	85.5	96.8	104.5	94.6	103.2	100.7	36.7	60.6	93.2	106.0	91.6	106.0
PRT	105.5	105.7	79.0	38.4	90.9	84.6	102.5	97.6	102.2	99.2	101.7	16.1	57.7	91.3	105.2	93.0	105.2
% change, 3DCRT vs. PRT	−3	−2	−1	**−71**	−1	−6	−1	−3	+3	+1	+7	**−100**	+56	+3	−3	+4	−3
% change, VMAT vs. PRT	−1	−1	−6	−40	+6	−6	0	−1	−1	0	+1	**−93**	−19	−4	−1	+4	−1

All values expressed as percentage of prescription dose. The percent change between modalities was calculated as ((PRT/other modality) × 100) − 100 for each patient; the median value for all patients is reported in the table. Boldface figures represent significant changes (*p* < 0.05). CTV: clinical target volume; PTV: planning target volume; i: ipsilateral; c: contralateral; ON: optic nerve; BS: brainstem; Csm: chiasm; SVZ: subventricular zone; Thal: thalamus; Hip: hippocampus; Pitu: pituitary; HT: hypothalamus; IT: infratentorial (normal) brain; ST: supratentorial (normal) brain; 3DCRT: three-dimensional conformal radiotherapy; VMAT: volumetric modulated arc therapy; PRT: proton radiotherapy.

**Table 3:** Median values for mean and maximum doses to target volumes and organs-at-risk by radiotherapy modality for suprasellar tumors.

Modality	CTV	PTV	iON	cON	BS	Csm	iSVZ	cSVZ	iThal	cThal	iHip	cHip	Pitu	HT	WB	IT	ST
	MEAN DOSE																
3DCRT	99.1	97.3	44.8	36.7	53.6	98.9	15.7	14.9	37.1	39.0	27.2	27.8	100.2	99.5	16.8	19.2	14.9
VMAT	99.6	97.8	44.1	41.1	40.7	97.9	18.5	17.5	45.2	50.1	32.7	37.1	99.6	99.4	16.0	19.5	15.4
PRT	99.7	98.7	39.2	39.6	26.7	96.1	14.9	14.1	40.2	38.9	20.2	20.7	99.9	100.1	9.5	6.0	10.1
% change, PRT vs. 3DCRT	0	+2	+4	−1	−33	−2	−1	0	−33	−35	−11	−22	+3	−1	**−35**	**−67**	**−29**
% change, PRT vs. VMAT	0	+1	−9	−9	−15	0	−21	−27	−10	−36	−21	−27	−10	−10	**−35**	**−64**	**−29**
	MAXIMUM DOSE																
3DCRT	103.4	103.4	99.6	99.6	99.5	101.5	94.3	92.3	99.3	97.1	74.6	81.1	101.5	101.9	102.9	102.9	101.9
VMAT	104.8	105.5	99.3	99.8	100.5	100.8	101.0	95.0	101.9	101.4	61.6	65.6	100.7	102.0	105.1	102.7	104.3
PRT	105.4	105.4	97.6	97.5	98.6	100.1	100.1	98.2	102.3	102.2	81.8	83.6	102.3	102.8	105.4	105.4	103.2
% change, 3DCRT vs. PRT	+2	+2	−2	−1	−2	−2	+1	+1	0	+3	+5	+3	+3	+5	+2	+2	+1
% change, VMAT vs. PRT	+1	0	0	0	−2	0	+2	+3	0	+1	+17	+9	+1	+1	0	+3	−2

All values expressed as percentage of prescription dose. The percent change between modalities was calculated as ((PRT/other modality) × 100) − 100 for each patient; the median value for all patients is reported in the table. Boldface figures represent significant changes (*p* < 0.05). CTV: clinical target volume; PTV: planning target volume; i: ipsilateral; c: contralateral; ON: optic nerve; BS: brainstem; Csm: chiasm; SVZ: subventricular zone; Thal: thalamus; Hip: hippocampus; Pitu: pituitary; HT: hypothalamus; IT: infratentorial (normal) brain; ST: supratentorial (normal) brain; 3DCRT: three-dimensional conformal radiotherapy; VMAT: volumetric modulated arc therapy; PRT: proton radiotherapy.

**Table 4:** Median values for mean and maximum doses to target volumes and organs-at-risk by radiotherapy modality for temporal tumors.

Modality	CTV	PTV	iON	cON	BS	Csm	iSVZ	cSVZ	iThal	cThal	iHip	cHip	Pitu	HT	WB	IT	ST
MEAN DOSE																	
3DCRT	99.3	98.6	71.4	27.1	66.4	86.9	91.4	41.4	97.7	65.2	96	47.6	78.7	86.7	43.6	54.0	55.1
VMAT	99.5	98.5	52.7	26.1	54.0	79.4	92.5	47.9	98.1	68.7	99.7	47.3	77.9	81.8	53.5	29.7	55.6
PRT	99.9	99.2	67.2	21.5	32.7	72.2	93.2	10.7	99.4	50.2	100.2	4.5	62.3	78.0	35.8	9.4	38.8
% change, PRT vs. 3DCRT	+1	0	−4	−24	−53	−15	+4	−22	+1	**−22**	1	**−90**	−74	−22	−12	−33	−26
% change, PRT vs. VMAT	0	+1	+15	−25	−41	−5	−1	−79	−1	**−27**	1	−93	−63	−20	−6	−32	−28
MAXIMUM DOSE																	
3DCRT	107.8	108.0	93.1	87.2	97.1	97.6	103.1	93.5	99.0	92.7	101.6	76.7	92.3	98.0	108.0	99.7	108.0
VMAT	107.2	107.2	89.5	70.5	99.2	95.4	103.2	88.3	104.5	99.4	103.5	73.2	90.0	101.5	107.2	102.5	107.2
PRT	107.2	107.2	90.7	69.9	101.4	95.2	104.5	90.0	103.5	102.0	103.7	52.7	86.3	102.3	107.2	103.4	107.2
% change, 3DCRT vs. PRT	0	0	−2	−15	+4	−1	+2	−3	+6	+10	+5	−21	+5	−1	+4	+6	−1
% change, VMAT vs. PRT	0	0	+2	−4	+2	+1	−1	−1	+1	+2	+1	−18	−7	+2	−1	+1	−1

All values expressed as percentage of prescription dose. The percent change between modalities was calculated as ((PRT/other modality) × 100) − 100 for each patient; the median value for all patients is reported in the table. Boldface figures represent significant changes (*p* < 0.05). CTV: clinical target volume; PTV: planning target volume; i: ipsilateral; c: contralateral; ON: optic nerve; BS: brainstem; Csm: chiasm; SVZ: subventricular zone; Thal: thalamus; Hip: hippocampus; Pitu: pituitary; HT: hypothalamus; IT: infratentorial (normal) brain; ST: supratentorial (normal) brain; 3DCRT: three-dimensional conformal radiotherapy; VMAT: volumetric modulated arc therapy; PRT: proton radiotherapy.

**Table 5:** Median values for mean and maximum doses to target volumes and organs-at-risk by radiotherapy modality for parietal tumors.

Modality	CTV	PTV	iON	cON	BS	Csm	iSVZ	cSVZ	iThal	cThal	iHip	cHip	Pitu	HT	WB	IT	ST
MEAN DOSE																	
3DCRT	100.8	100.3	5.3	4.8	35.4	36.4	79.0	34.9	77.1	44.1	59.8	24.5	12.1	39.7	49.5	28.5	51.1
VMAT	100.0	99.4	9.2	8.2	16.7	27.8	69.1	33.0	87.2	46.6	24.1	60.2	12.3	36.5	42.5	5.8	46.7
PRT	99.9	99.2	0.1	0.0	1.8	3.1	58.5	3.1	61.5	5.0	39.5	0.3	0.1	7.9	24.9	0.5	27.8
%change, PRT vs. 3DCRT	−1	−1	**−100**	**−100**	**−90**	**−94**	−31	**−92**	−48	**−92**	−34	**−99**	**−99**	**−99**	−55	**−98**	−54
%change, PRT vs. VMAT	0	0	**−100**	**−100**	**−83**	**−93**	−17	**−89**	−21	**−87**	+86	**−97**	**−100**	**−86**	−41	**−98**	−38
MAXIMUM DOSE																	
3DCRT	107.2	107.3	17.7	12.7	83.2	68.5	102.5	64.2	99.6	79.6	100.8	50.8	25.4	66.4	107.3	79.6	107.3
VMAT	104.9	104.9	16.5	17.6	79.4	45.1	103.0	53.8	102.1	74.3	103.0	47.7	17.4	60.7	104.9	67.7	104.9
PRT	105.6	105.6	1.2	0.2	46.3	34.7	102.9	14.7	103.3	50.6	102.1	4.2	0.8	49.5	105.6	25.7	105.6
% change, 3DCRT vs. PRT	+1	+1	**−96**	**−93**	−46	**−77**	+1	−75	+3	−53	+3	**−82**	**−92**	−38	+1	−79	−2
% change, VMAT vs. PRT	+1	+1	**−95**	**−98**	−31	−96	+1	−71	+1	−35	−1	**−82**	**−96**	−34	+1	−76	+1

All values expressed as percentage of prescription dose. The percent change between modalities was calculated as ((PRT/other modality) × 100) − 100 for each patient; the median value for all patients is reported in the table. Boldface figures represent significant changes (*p* < 0.05). CTV: clinical target volume; PTV: planning target volume; i: ipsilateral; c: contralateral; ON: optic nerve; BS: brainstem; Csm: chiasm; SVZ: subventricular zone; Thal: thalamus; Hip: hippocampus; Pitu: pituitary; HT: hypothalamus; IT: infratentorial (normal) brain; ST: supratentorial (normal) brain; 3DCRT: three-dimensional conformal radiotherapy; VMAT: volumetric modulated arc therapy; PRT: proton radiotherapy.

**Table 6:** Median values for mean and maximum doses to target volumes and organs-at-risk by radiotherapy modality for tumors of the posterior cranial fossa.

Modality	CTV	PTV	iON	cON	BS	Csm	iSVZ	cSVZ	iThal	cThal	iHip	cHip	Pitu	HT	WB	IT	ST
MEAN DOSE																	
3DCRT	98.7	97.8	4.5	6.1	54.5	11.7	7.2	3.6	2.7	2.6	30.8	25.9	18.7	10.6	13.5	52.0	8.0
VMAT	99.8	99.3	10.6	8.5	57.8	15.1	5.1	4.0	3.5	3.6	32.4	28.3	26.4	16.1	13.5	56.1	7.0
PRT	99.7	98.6	0.0	0.0	58.6	0.6	10.3	5.2	7.8	7.1	42.5	27.5	0.9	0.9	8.6	43.3	6.0
%change, PRT vs. 3DCRT	+1	0	**−100**	**−100**	0	**−82**	−39	−59	−32	−35	−16	−16	**−86**	**−72**	−27	−12	−45
%change, PRT vs. VMAT	0	−1	**−100**	**−100**	−2	**−84**	−28	−44	−35	−43	−35	−22	**−95**	−77	−26	−18	−30
MAXIMUM DOSE																	
3DCRT	103.2	103.2	11.0	21.3	99.6	25.6	60.1	32.3	6.3	6.1	91.1	64.8	25.6	29.9	103.2	103.1	98.3
VMAT	102.9	103.3	24.1	20.1	101.4	29.6	49.6	32.4	13.5	15.8	87.6	54.5	30.5	34.2	103.3	102.7	102.0
PRT	104.9	104.9	0.3	0.3	101.6	3.0	40.5	32.2	29.6	29.3	86.8	64.2	2.2	4.3	104.9	104.9	102.4
% change, 3DCRT vs. PRT	+1	+1	**−94**	**−96**	+1	−42	−35	−28	20	4	+2	+1	−81	−45	+1	+1	+2
% change, VMAT vs. PRT	0	0	**−98**	**−98**	0	−51	−12	−8	14	12	+1	+5	−91	−61	0	+1	−2

All values expressed as percentage of prescription dose. The percent change between modalities was calculated as ((PRT/other modality) × 100) − 100 for each patient; the median value for all patients is reported in the table. CTV: clinical target volume; PTV: planning target volume; i: ipsilateral; c: contralateral; ON: optic nerve; BS: brainstem; Csm: chiasm; SVZ: subventricular zone; Thal: thalamus; Hip: hippocampus; Pitu: pituitary; HT: hypothalamus; IT: infratentorial (normal) brain; ST: supratentorial (normal) brain; 3DCRT: three-dimensional conformal radiotherapy; VMAT: volumetric modulated arc therapy; PRT: proton radiotherapy.

**Table 7:** Comparison of target volume coverage.

	3D-Photon RT	VMAT RT	Proton RT
**frontal**			
D_max_ (in %)	108.67 ± 1.58	107.39 ± 2.12	105.54 ± 1.58 *^,^ **^†^**
D_mean_ (in %)	100.94 ± 1.16	99.80 ± 0.58	99.83 ± 0.20
D_min_ (in %)	84.14 ± 13.42	71.17 ± 14.79	82.82 ± 7.85
HI (in %)	8.80 ± 4.48	5.69 ± 5.00	4.17± 0.84
IC (in %)	0.27 ± 0.18	0.43± 0.15	**0.24 ± 0.09** **^†^**
**temporal**			
D_max_ (in %)	107.84 ± 2.38	107.23 ± 2.01	106.56 ± 2.45
D_mean_ (in %)	99.27 ± 1.37	99.45 ± 0.40	99.78 ± 1.39
D_min_ (in %)	86.57 ± 10.75	74.48 ± 21.38	84.90 ± 12.21
HI (in %)	9.94 ± 5.61	8.97 ± 3.66	4.67 ± 2.03
IC (in %)	0.19 ± 0.11	0.32 ± 0.22	0.22 ± 0.13
**parietal**			
D_max_ (in %)	107.23 ± 2.20	104.92 ± 1.78	105.58 ± 1.96
D_mean_ (in %)	100.76 ± 1.12	100.01 ± 0.24	99.92 ± 0.07
D_min_ (in %)	92.69 ± 10.38	84.90 ± 12.21	90.90 ± 4.09
HI (in %)	6.62 ± 4.18	3.95 ± 2.06	3.87 ± 1.10
IC (in %)	0.15 ± 0.10	0.13 ± 0.09	0.16 ± 0.05
**suprasellar**			
D_max_ (in %)	103.40 ± 2.65	104.85 ± 2.02	105.44 ± 2.05
D_mean_ (in %)	99.09 ± 0.81	99.62 ± 0.31	99.66 ± 0.37
D_min_ (in %)	79.81 ± 8.10	76.05 ± 12.94	91.48 ± 4.74 *^,^ **^†^**
HI (in %)	7.69 ± 3.47	5.32 ± 2.06	5.62 ± 2.50
IC (in %)	0.24 ± 0.08	0.29 ± 0.15	0.15 ± 0.06 *^,^ **^†^**
**posterior cranial fossa**			
D_max_ (in %)	103.23 ± 2.29	102.9 ± 4.12	104.88 ± 2.33
D_mean_ (in %)	98.69 ± 1.94	99.78 ± 1.57	99.78 ± 1.39
D_min_ (in %)	89.16 ± 6.29	92.58 ± 14.65	91.63 ± 5.78
HI (in %)	6.92 ± 4.65	4.24 ± 12.33	5.24 ± 1.91
IC (in %)	0.14 ± 0.08	0.15 ± 0.17	0.15 ± 0.06

D_max_ = maximum dose to the CTV; D_mean_ = average dose to the CTV; D_min_ = minimum dose to the CTV; HI (homogeneity index) = (D5% − D95%)/prescribed dose × 100; IC (inhomogeneity coefficient) = (D_max_ − D_min_)/D_mean_; values are given as mean values with standard deviations. Boldface figures represent significant changes (*p* < 0.05). ^†^ Statistically significant (IMPT/PRT vs. VMAT); * Statistically significant (IMPT/PRT vs. 3DCRT). RT: radiation therapy; IMPT: intensity-modulated proton therapy; PRT: proton radiotherapy; VMAT: volumetric-modulated arc therapy; 3DCRT: three-dimensional conformal radiotherapy.
